# Comparative Analysis of Dental Treatment Accuracy Using 2.5× Magnification Loupes Versus the Naked Eye: A Randomized Clinical Trial

**DOI:** 10.7759/cureus.108204

**Published:** 2026-05-03

**Authors:** Talacheeru Rohit, Sravya Sri Prudhvi, Sreenivas Nagarakanti, Sandhya B, Hareesh Bandi, Kunapareddy Madhu Hasini

**Affiliations:** 1 Periodontology, Narayana Dental College and Hospital, Nellore, IND

**Keywords:** dental loupes, dental scaling, magnification, randomized controlled trial, visual acuity

## Abstract

Background

Magnification systems such as dental loupes have been increasingly incorporated into clinical dental practice to improve visualization, precision, and ergonomics. However, evidence evaluating their effectiveness during routine periodontal procedures performed by dental interns remains limited. The present study aimed to assess the effect of 2.5× dental magnification loupes on psychomotor skill acquisition and clinical treatment outcomes during ultrasonic scaling.

Methodology

This randomized clinical trial was conducted in the Department of Periodontology at Narayana Dental College and Hospital. A total of 20 patients requiring routine scaling were randomly assigned to either a magnification group (2.5× dental loupes) or a control group (conventional scaling without magnification), with 10 participants in each group. Ultrasonic scaling procedures were performed by dental interns. Clinical outcomes were evaluated using the Calculus Surface Index (CSI) component of the Oral Hygiene Index before and after scaling. Operator fatigue, treatment duration, and postural comfort were also assessed. Statistical analysis was performed using SPSS version 25, and a p-value <0.05 was considered statistically significant.

Results

Both groups demonstrated significant reductions in CSI scores following ultrasonic scaling, with mean reductions of 1.40 ± 0.52 in the control group and 1.90 ± 0.88 in the magnification group (p < 0.001). Baseline CSI scores were comparable between the groups (p = 0.913). However, the magnification group showed a greater reduction in CSI scores after treatment compared with the control group (p < 0.05). Postural comfort was significantly better among operators using magnification loupes (p = 0.012). Nevertheless, the magnification group demonstrated slightly higher fatigue scores and longer procedure duration.

Conclusions

The use of 2.5× dental magnification loupes during ultrasonic scaling may enhance calculus detection and improve clinical outcomes while promoting better operator ergonomics. Incorporating magnification-assisted techniques into dental training may contribute to improved psychomotor skills and clinical performance.

## Introduction

In recent years, dentistry has undergone a significant transformation with the integration of advanced visualization technologies aimed at improving diagnostic accuracy and treatment precision. Dental procedures require a high level of visual acuity and refined motor skills to perform delicate clinical tasks effectively. Magnification is one approach increasingly adopted in modern dentistry to enhance visual perception and improve clinical performance [1].

Magnification systems, such as dental loupes and operating microscopes, enhance visualization, procedural precision, and clinician ergonomics. These technologies have become essential components of modern micro-dentistry, supporting greater accuracy and potentially contributing to improved clinical outcomes [2].

Among the available magnification systems, dental loupes are the most used due to their portability, affordability, and availability in various magnification levels, typically ranging from 1.5× to 10× [3]. In routine dental procedures, magnification levels between 2.0× and 3.5× are most utilized, whereas magnifications of 4.0× or higher are considered high-power and are generally reserved for procedures requiring enhanced detail, such as endodontic and periodontal microsurgeries [4]. The use of magnification in dentistry provides three primary benefits: improved visual clarity, increased precision during clinical procedures, and enhanced ergonomics through the promotion of an upright working posture [3].

Ergonomic loupes represent an advancement in magnification technology and are specifically designed to support improved posture through optimized declination angles and lightweight construction. These features help reduce musculoskeletal strain and improve operator comfort while maintaining high levels of precision during procedures that require detailed visualization, such as periodontal microsurgery [5]. Consequently, these advantages may not only contribute to improved treatment outcomes but also support the long-term occupational health and sustainability of dental practitioners.

The primary objective of periodontal therapy is the removal of bacterial deposits from tooth surfaces and the subgingival environment through procedures such as scaling and root planing (SRP). Both manual and ultrasonic instruments are widely used for this purpose and have demonstrated effectiveness in improving clinical periodontal parameters [6]. Conventional scaling remains the gold standard in periodontal therapy and is considered a prerequisite for successful periodontal treatment outcomes [7].

The use of magnification during periodontal procedures may further enhance clinical performance by improving visual acuity, facilitating better detection of calculus deposits, and improving color contrast between calculus and the surrounding tooth structure. Additionally, magnification may enhance tactile perception during instrumentation while promoting improved operator posture [8].

With the increasing emphasis on incorporating magnification techniques into dental education and clinical training, it is important to evaluate their actual clinical benefits. Therefore, the present randomized clinical trial aims to assess the effect of 2.5× dental magnification loupes on psychomotor skill acquisition and clinical treatment outcomes during ultrasonic scaling performed by dental interns.

## Materials and methods

Ethical approval

This study was approved by the Institutional Ethical Committee (reference number: EC/NEW/INST/2024/4166) of Narayana Dental College and Hospital, Nellore, Andhra Pradesh, India, and was registered with the Clinical Trials Registry - India (registration number: CTRI/2025/12/099753). The study was conducted from January 8, 2026, to February 7, 2026, which was within the approved study period granted by the ethics committee.

Participants

This study was designed as a randomized clinical trial conducted in the Department of Periodontology, Narayana Dental College and Hospital, Nellore, Andhra Pradesh, India. Participants included patients visiting the outpatient Department of Periodontology who required routine scaling procedures. Dental interns posted in the department served as the operators performing the scaling procedures under magnification or without magnification.

Inclusion criteria

The study included individuals aged 25-55 years. Those with the presence of supragingival and/or subgingival calculus were considered for inclusion. Participants needed to be able to understand and sign a written informed consent form. Participants needed to have at least 20 teeth. Participants needed not to have any systemic disease or conditions. Patients had to be diagnosed with biofilm-induced gingivitis with supragingival or minimal subgingival calculus deposits.

Exclusion criteria

Individuals with periodontitis (clinical attachment loss >3 mm), a history of periodontal therapy in the past six months, smokers, pregnant/lactating women, and those not meeting the inclusion criteria were excluded.

Sampling method and sample size

The sample size was determined considering a significance level of 5% and a power of 80%. A total of 10 participants per group was calculated to be sufficient to detect a clinically meaningful difference between groups. Participants were recruited from patients attending the outpatient Department of Periodontology at Narayana Dental College and Hospital based on the predefined inclusion and exclusion criteria. Ultrasonic scaling procedures were performed by 10 dental interns during their clinical postings in the department. Each intern performed scaling on two participants, one from the test group and one from the control group, to minimize operator-related variability. All participants were clinically evaluated before and after scaling using the Calculus Surface Index (CSI), assessed through visual and tactile examination with a mouth mirror and explorer, by a calibrated periodontist who was blinded to the treatment allocation. In the test group, ultrasonic scaling was performed to remove supragingival and subgingival calculus under magnification using 2.5× dental loupes. In the control group, ultrasonic scaling was performed to remove supragingival and subgingival calculus under conventional vision without magnification.

Ultrasonic scaling protocol

Ultrasonic scaling was performed using a standardized protocol for all participants. A piezoelectric ultrasonic scaler with a universal scaling tip was used at a consistent medium power setting throughout the study. Each quadrant was instrumented for a standardized duration under adequate illumination. Operators were instructed to maintain a consistent working position and patient positioning in accordance with recommended ergonomic guidelines.

Randomization and allocation concealment

**Figure 1:**
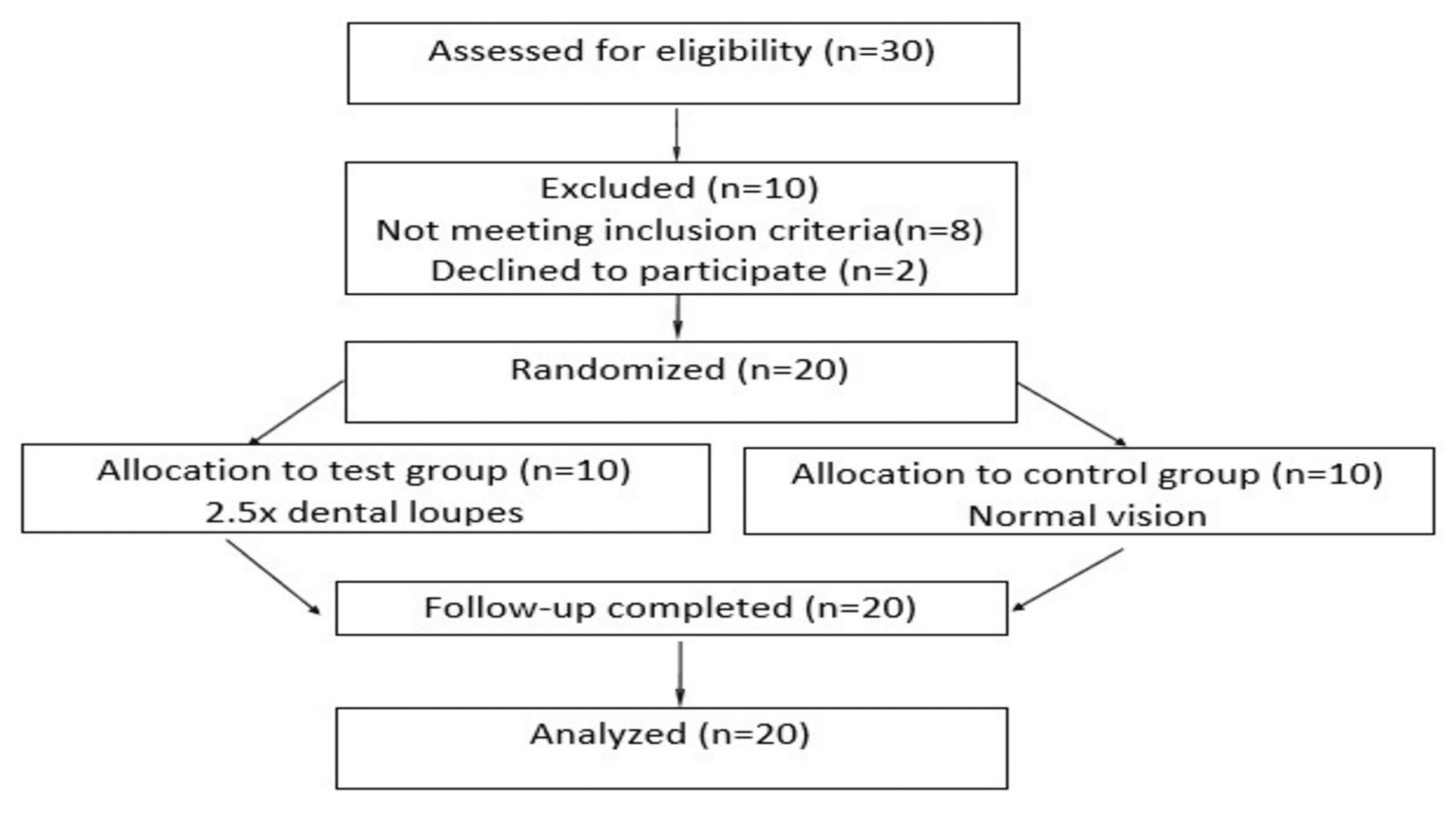
CONSORT flow diagram of participant recruitment, randomization, allocation, follow-up, and analysis. A total of 30 participants were assessed for eligibility, of whom 10 were excluded (eight did not meet the inclusion criteria, and two declined to participate). The remaining 20 participants were randomized into two groups: the test group (n = 10), in which scaling was performed using 2.5× magnification dental loupes, and the control group (n = 10), where scaling was performed under normal vision without magnification. All participants completed follow-up, and data from all 20 participants were included in the final analysis.

**Figure 2:**
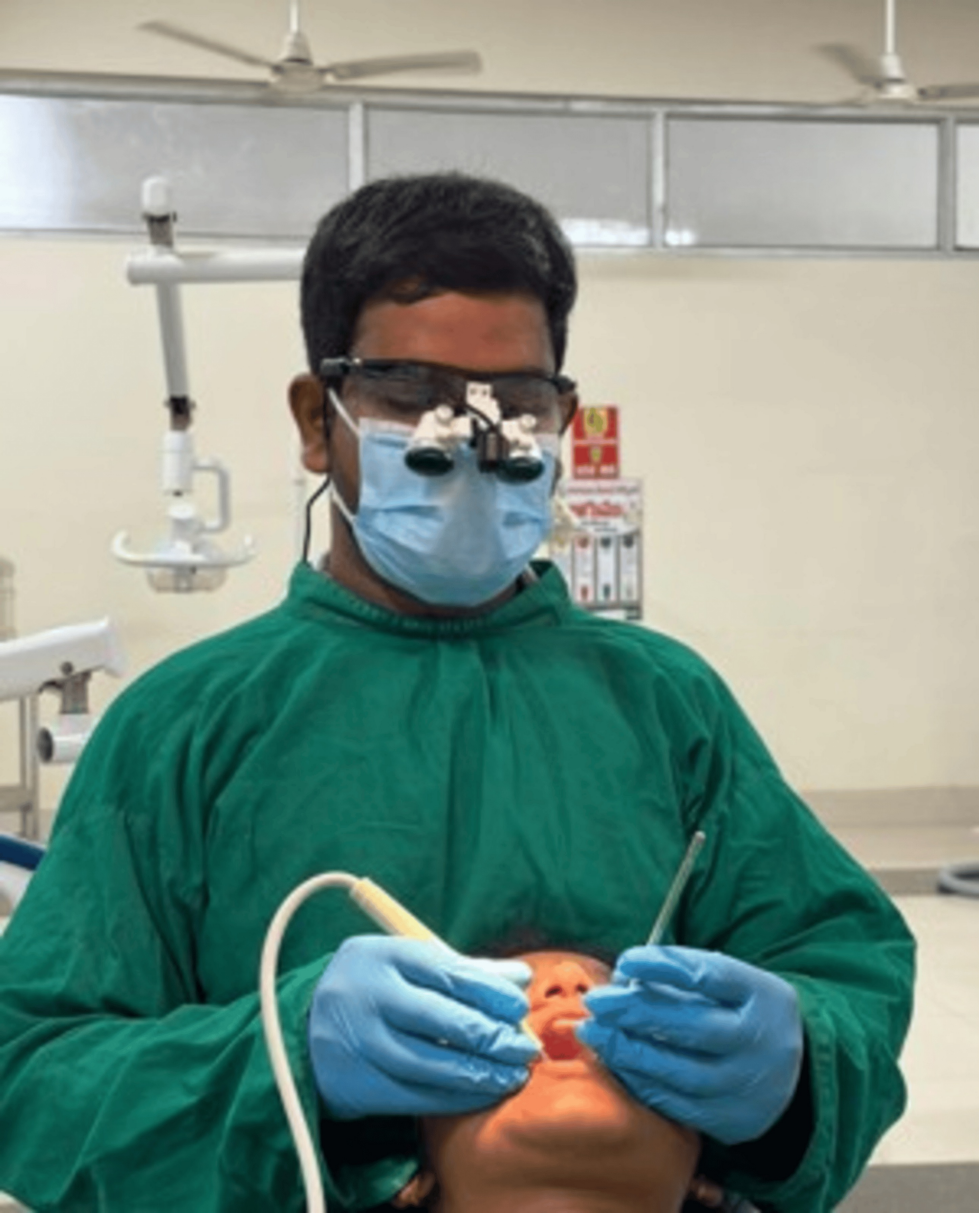
Ultrasonic scaling performed under magnification.

**Figure 3:**
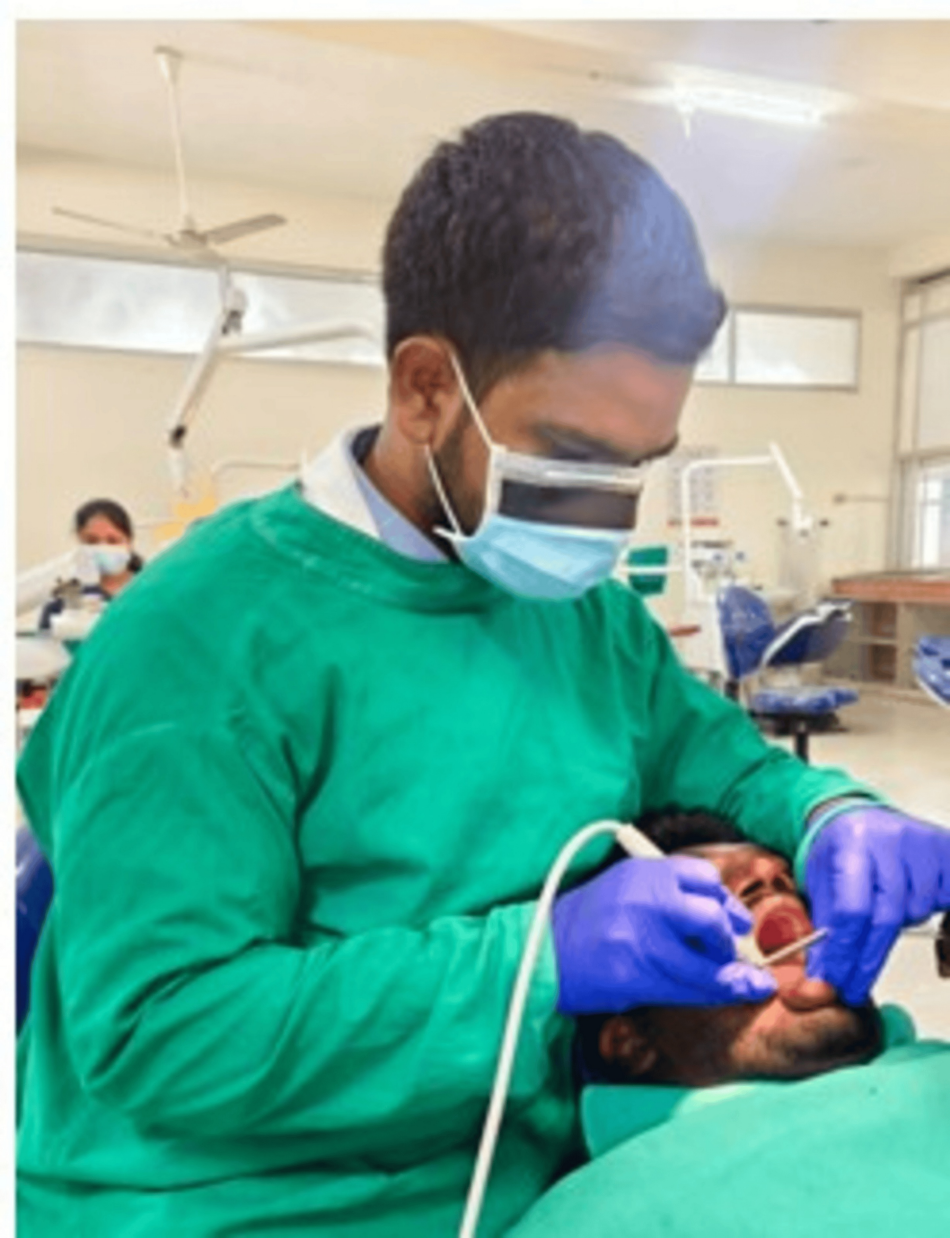
Ultrasonic scaling performed without magnification.

Participants were randomly assigned to either the test group or the control group using a computer-generated randomization sequence prepared before the initiation of the study to ensure unbiased allocation. The randomization sequence was concealed using sealed, opaque envelopes to maintain allocation concealment. Each envelope was opened only at the time of intervention, after participant enrollment, to determine the group assignment. The participant recruitment and allocation process is illustrated in the CONSORT flow diagram (Figure 1). Participants were allocated to either the magnification-assisted scaling group (test group) depicted in Figure 2 or the conventional ultrasonic scaling group (control group) depicted in Figure 3.

Operator training and calibration

Before the study, all participating interns underwent standardized training sessions to ensure uniformity in scaling technique. Calibration exercises were conducted under supervision to achieve consistency in instrumentation and adherence to the study protocol.

Blinding

The present study was designed as a single-blind randomized clinical trial, wherein the outcome assessor was blinded to the group allocation. Due to the nature of the intervention, operator blinding was not feasible.

Outcome measures

The primary objective was to assess treatment accuracy, measured using the CSI scores before and after scaling. The secondary objectives included evaluation of operator ergonomics (posture), fatigue, and treatment duration on a Visual Analogue Scale (VAS) from one to 10. In this context, psychomotor performance was considered in terms of the efficiency and precision of instrumentation during scaling procedures. It was hypothesized that the use of 2.5× magnification loupes would result in improved treatment accuracy, better ergonomics, and reduced operator fatigue compared to procedures performed without magnification.

Statistical analysis

The collected data were compiled using Microsoft Excel (Microsoft Corp., Redmond, WA, USA) and subsequently analysed with SPSS software, version 25 (IBM Corp., Armonk, NY, USA). To assess differences within groups for continuous variables, the paired t-test was employed. Comparisons between groups were analyzed using independent t-tests for continuous variables and chi-square tests for categorical variables. A p-value of less than 0.05 was considered statistically significant for all analyses.

## Results

A total of 20 participants were included in the study and completed the intervention. Intergroup comparisons of operator fatigue and treatment duration are presented in Table 1. The mean fatigue score was higher in the magnification group (7.40 ± 0.70) compared with the control group (6.50 ± 0.71), showing a statistically significant difference (p < 0.05). The duration of the scaling procedure was also significantly greater in the magnification group (35.00 ± 5.77 minutes) compared with the control group (28.50 ± 3.37 minutes). Clinically, this increase suggests that the use of magnification may require additional time for more precise and meticulous instrumentation, particularly during the initial phase of operator adaptation.

**Table 1:** Intergroup comparison of operator fatigue and procedure duration. Data are presented as mean ± standard deviation (SD). Intergroup comparisons were performed using an independent t-test. A p-value <0.05 was considered statistically significant. * indicates statistically significant difference.

Parameter	Group	Mean ± SD	Mean difference	95% CI	P-value
Fatigue score	Control	6.50 ± 0.71	-0.90	-1.56 to 0.24	0.012*
	Test (magnification)	7.40 ± 0.70			
Duration of procedure (minutes)	Control	28.50 ± 3.37	-6.50	-10.94 to -2.06	0.008*
	Test (magnification)	35.00 ± 5.77			

Postural comfort scores differed significantly between groups (Table 2). Operators using magnification loupes reported lower discomfort scores compared with those performing scaling under conventional vision (p = 0.012), indicating improved ergonomic posture with the use of magnification.

**Table 2:** Intergroup comparison of postural comfort. Data are presented as mean ± standard deviation (SD). Intergroup comparison was performed using an independent t-test. A p-value <0.05 was considered statistically significant. * indicates statistically significant difference.

Parameter	Group	Mean ± SD	Mean difference	95% CI	P-value
Postural comfort score	Test (magnification)	6.10 ± 0.57	-1.20	-1.93–0.47	0.012*
	Control	7.30 ± 0.95			

Intragroup analysis revealed a significant reduction in CSI scores following scaling in both groups (Table 3). However, the reduction was greater in the magnification group (1.90 ± 0.88) compared with the control group (1.40 ± 0.52), suggesting that the use of magnification loupes may enhance calculus removal during ultrasonic scaling.

**Table 3:** Intragroup comparison of Calculus Surface Index (CSI) (pre- and post-scaling). Data are presented as mean ± standard deviation (SD). Intragroup comparison between pre- and post-scaling values was performed using a paired t-test. A p-value <0.05 was considered statistically significant. * indicates statistically significant difference.

Group	Mean reduction (mean ± SD)	Mean difference	95% CI	t-value	P-value
Control	1.40 ± 0.52	0.16	1.03–1.77	8.57	<0.001*
Test (magnification)	1.90 ± 0.88	0.28	1.27–2.53	6.86	<0.001*

Intergroup comparison of the CSI is shown in Table 4. At baseline, no statistically significant difference was observed between the two groups (p = 0.913), indicating comparable clinical conditions before treatment. Following scaling, the magnification group demonstrated a greater reduction in CSI compared with the control group, and this difference was statistically significant (p < 0.05).

**Table 4:** Intergroup comparison of Calculus Surface Index (CSI). Data are presented as mean ± standard deviation (SD). Intergroup comparisons were performed using an independent t-test. A p-value <0.05 was considered statistically significant. * indicates statistically significant difference.

Time point	Group	Mean ± SD	Mean difference	95% CI	P-value
Pre-scaling	Control	2.30 ± 0.82	0.20	-0.60–1.00	0.913
	Test	2.10 ± 0.88			
Post-scaling	Control	0.90 ± 0.99	0.70	-0.08–1.22	0.009*
	Test	0.20 ± 0.63			

## Discussion

Plaque-induced gingival inflammation primarily develops because of the accumulation of microbial biofilms and calculus deposits on tooth surfaces. These deposits harbor microorganisms capable of releasing endotoxins and inflammatory mediators that contribute to the destruction of periodontal supporting tissues. Effective management of periodontal disease, therefore, relies on the mechanical disruption and removal of these deposits through SRP. Although SRP remains the primary nonsurgical treatment modality, complete removal of subgingival deposits can be challenging due to restricted access and limited visibility during instrumentation, particularly in deeper periodontal pockets and on anatomically complex root surfaces [9].

Magnification devices such as dental loupes have been introduced in clinical dentistry to improve visualization of the operative field. By enlarging the working area and enhancing visual clarity, magnification may allow clinicians to perform more precise instrumentation and detect residual calculus deposits more effectively. Previous studies evaluating scaling procedures performed with and without magnification have reported improvements in periodontal parameters with both techniques [8,10]. Sonika et al. conducted a randomized clinical trial comparing SRP performed with and without magnification loupes and reported improvements in periodontal outcomes in both groups, although the differences were not statistically significant [8]. Similarly, Parvez and Manjunath observed improvements following both conventional and magnification-assisted scaling procedures [10].

However, the present study differs in that it demonstrated a statistically significant greater reduction in CSI scores in the magnification group compared with the control group. Additionally, unlike previous studies that primarily focused on clinical periodontal parameters, the present study also evaluated operator-related outcomes such as postural comfort, fatigue, and procedure duration, thereby providing a more comprehensive assessment of the clinical and ergonomic impact of magnification-assisted scaling.

Improved visualization during periodontal instrumentation may also contribute to more effective root surface debridement. Studies evaluating root surface morphology following scaling procedures have suggested that magnification can assist clinicians in identifying residual deposits and performing more thorough instrumentation [11]. The use of high-magnification optical systems has also been reported to enhance the detection of residual calculus during prophylaxis and periodontal therapy [12].

In the present study, both the control and magnification groups demonstrated significant reductions in CSI scores following ultrasonic scaling. These findings support earlier evidence demonstrating that mechanical periodontal debridement is effective in reducing plaque and calculus accumulation and controlling periodontal inflammation [9]. However, the reduction in CSI scores observed in the magnification group was greater than that observed in the control group. This suggests that improved visualization provided by magnification loupes may facilitate better identification and removal of calculus deposits during periodontal instrumentation.

Apart from clinical effectiveness, ergonomic considerations represent an important aspect of dental practice. Dental professionals frequently maintain static and forward-leaning postures during clinical procedures, which may contribute to musculoskeletal discomfort over time. Previous studies have reported that magnification systems can encourage a more upright working posture and improve ergonomic positioning during dental procedures [13,14]. Carpentier et al. demonstrated that dental students using magnification loupes during preclinical procedures adopted more favorable postural positions compared with those working without magnification [13]. Similarly, Maillet et al. reported improvements in operator posture among dental hygiene students using magnification loupes [14]. Branson et al. also observed that magnification lenses positively influenced operator posture during dental procedures [15]. Ludwig et al. further reported reduced neck and trunk flexion among dental hygienists performing scaling procedures with magnification loupes [16].

The findings of the present study are consistent with these observations. Operators who performed scaling procedures using magnification loupes reported significantly lower postural discomfort scores compared with those in the control group. Improved ergonomic positioning may help clinicians maintain a more neutral working posture during prolonged procedures. This is particularly relevant because musculoskeletal disorders are commonly reported among dental professionals due to repetitive movements and sustained postural strain [17,18]. Ergonomic principles in dentistry emphasize maintaining appropriate posture and using visual aids such as magnification systems to minimize occupational stress [19].

Despite these advantages, the magnification group demonstrated slightly higher fatigue scores and required a longer duration to complete the scaling procedures. A possible explanation for this observation may be the learning curve associated with the use of magnification devices. Dental interns who are newly introduced to loupe-assisted procedures may initially require additional time to adapt to the magnified field and coordinate hand movements effectively. Previous studies have suggested that clinicians may experience temporary increases in treatment duration while adjusting to magnification-assisted techniques [17]. With continued experience and routine use, however, the efficiency of magnification-assisted procedures is likely to improve.

Overall, the findings of the present study indicate that the use of magnification loupes can improve the precision of periodontal instrumentation while also supporting better ergonomic posture among clinicians. Although a slight increase in treatment duration was observed, the benefits of enhanced visualization and improved operator comfort highlight the potential value of magnification systems in periodontal practice. Incorporating magnification-assisted techniques into dental education and clinical training programs may therefore contribute to the development of improved clinical skills and healthier ergonomic practices among dental practitioners performing nonsurgical periodontal therapy.

Clinical implications

The findings of the present study highlight the potential benefits of incorporating magnification loupes into routine periodontal instrumentation and dental training programs. Improved visualization during ultrasonic scaling may facilitate more effective detection and removal of calculus deposits, thereby enhancing treatment precision. In addition, the use of magnification loupes may promote improved ergonomic posture among clinicians, which can help reduce musculoskeletal strain commonly experienced during dental procedures. The integration of magnification-assisted techniques in undergraduate clinical training may also aid in the development of better psychomotor skills and clinical efficiency among dental interns.

Limitations

Despite the encouraging findings, certain limitations of the present study should be acknowledged. The study was conducted with a relatively small sample size and within a single institutional setting, which may limit the generalizability of the results. Additionally, the procedures were performed by dental interns, and variations in operator experience may have influenced procedural efficiency and fatigue levels. The study also evaluated short-term clinical outcomes immediately following scaling, without assessing long-term periodontal changes. Future studies with larger sample sizes, multicenter participation, and extended follow-up periods are required to further validate the clinical benefits of magnification-assisted periodontal instrumentation.

## Conclusions

Within the limitations of the present study, ultrasonic scaling performed with and without magnification loupes resulted in significant reductions in CSI scores, indicating the effectiveness of both techniques in reducing calculus accumulation. However, the use of magnification loupes demonstrated greater improvements in calculus removal and was associated with significantly lower postural discomfort among operators. These findings suggest that magnification systems may enhance visualization during periodontal instrumentation and promote improved ergonomic posture during clinical procedures. Although slightly longer treatment durations were observed when magnification was used, this may be attributed to the learning curve associated with loupe-assisted techniques. Overall, magnification loupes may serve as a valuable adjunct in periodontal instrumentation and dental training, potentially improving both treatment precision and operator ergonomics.
